# Autophagy: A new strategy for host-directed therapy of tuberculosis

**DOI:** 10.1080/21505594.2018.1536598

**Published:** 2018-11-02

**Authors:** Seungwha Paik, Jin Kyung Kim, Chaeuk Chung, Eun-Kyeong Jo

**Affiliations:** a Department of Microbiology and Infection Control Convergence Research Center, Chungnam National University School of Medicine, Daejeon, Korea; b Department of Medical Science, Chungnam National University School of Medicine, Daejeon, Korea; c Division of Pulmonary and Critical Care, Department of Internal Medicine, Chungnam National University School of Medicine, Daejeon, Korea

**Keywords:** *Mycobacterium tuberculosis*, xenophagy, LC3-associated phagocytosis, host-directed therapy, innate immunity, tuberculosis

## Abstract

Tuberculosis (TB), which is primarily caused by the major etiologic agent *Mycobacterium tuberculosis* (Mtb), remains a serious infectious disease worldwide. Recently, much effort has been made to develop novel/improved therapies by modulating host responses to TB (i.e., host-directed therapy). Autophagy is an intracellular catabolic process that helps maintain homeostasis or the removal of invading pathogens via a lysosomal degradation process. The activation of autophagy by diverse drugs or agents may represent a promising treatment strategy against Mtb infection, even to drug-resistant strains. Important mediators of autophagy activation include vitamin D receptor signaling, the AMP-activated protein kinase pathway, sirtuin 1 activation, and nuclear receptors. High-throughput approaches have identified numerous natural and synthetic compounds that enhance antimicrobial defense against Mtb infection through autophagy. In this review, we discuss the current knowledge of, advancements in, and perspectives on new therapeutic strategies targeting autophagy against TB. Understanding the mechanisms and key players involved in modulating antibacterial autophagy will provide innovative improvements in anti-TB therapy via an autophagy-targeting approach.

**Abbreviations**: TB: Tuberculosis; Mtb: *Mycobacterium tuberculosis*; HDT: host-directed therapy; MDR: multidrug resistant; XDR: extensively drug resistant; LAP: LC3-associated phagocytosis; ROS: reactive oxygen species; VDR: vitamin D receptor; TFEB: transcription factor EB; ERRα: estrogen-related receptor α; PGC1α: PPARγ coactivator-1 α

## Introduction

The autophagy process plays a fundamentally important basal housekeeping function in diverse physiological conditions. Autophagy activation is required for the maintenance of cellular homeostasis and survival by providing energy building blocks during a variety of stresses via the cell autonomous digestion of intracytoplasmic cargo (i.e., large macromolecular aggregates and damaged organelles). Autophagy is also critical for the regulation of a wide range of immune responses including innate immunity, inflammation, and macrophage antibacterial defenses [1,2].

Human TB is an infectious disease with high morbidity and mortality and remains a global threat. The standard treatment for TB is a regimen of frontline combination chemotherapy with multiple antibiotics for at least 6 months. Current anti-TB therapy has many limitations such as prolonged treatment duration, drug toxicity, and potential risk for the development of drug-resistant strains if patients are noncompliant. Therefore, there is an urgent need to develop new therapeutic drugs to control infection more effectively [3]. Host-directed therapy (HDT) may be useful for fighting bacterial infections to improve the treatment efficiency for TB [4,5]. In addition, HDT-TB may be applicable to the treatment of multidrug (MDR)- or extensively drug (XDR)-resistant TB via therapeutic targeting of numerous clinically relevant biological pathways in hosts [6,7]. Based on the modulation of pathological or protective responses in hosts, a variety of components/pathways of the immune system are central players that contribute to therapeutic targets for HDT against TB. Candidate targets include modulators of pathologic inflammation, antimicrobial effectors, and drugs/reagents for the maintenance of homeostasis [3,7]. Because autophagy is critical for maintaining intracellular homeostasis and acts as a crucial immune arm, autophagy modulators/molecules could represent promising candidates in the context of HDT against TB with or without adjunctive agents for standard therapeutics [4,8].

In this review, we focus on current advances in the identification of autophagy-activating agents exhibiting antibacterial activity as potential therapeutics to eradicate Mtb infection. More attention should be paid to the identification of key players and mechanisms by which autophagy-activating agents target bacteria to enhance antimicrobial responses. Finally, we discuss the challenges and perspectives of autophagy-adjunctive therapeutics for their clinical use.

## Overview of autophagy

**Figure 1.**
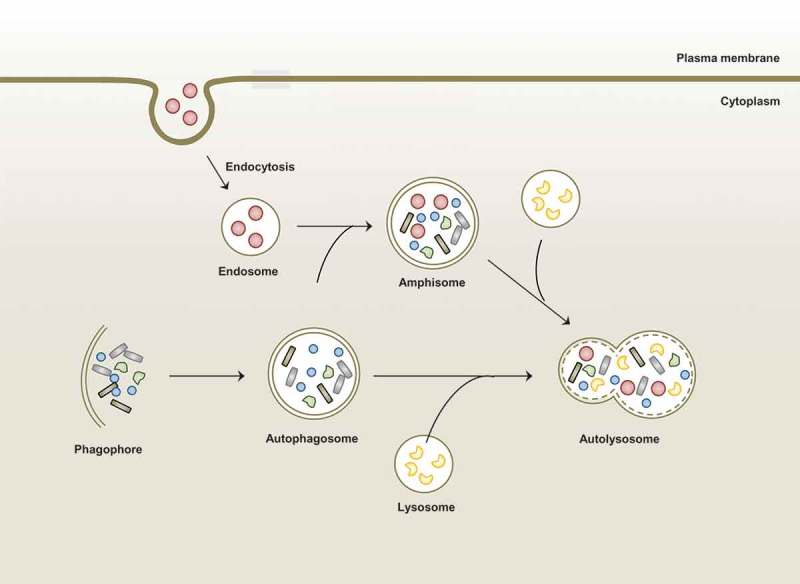
Overview of macroautophagy. Macroautophagy is one of autophagic process in which is induced by stimuli or stress such as starvation and infection. Autophagy sequesters aggregated proteins, damaged organelles, and microbes from cytoplasm through formation of double-membraned structure (autophagosome). These autophagosomes fuse with lysosome or endosome that contains endocytic compartments to form amphisome or autolysosome. Finally, cargos are degraded in autolysosome for the maintenance of cellular homeostasis.

Autophagy (herein, macroautophagy) is an intracellular homeostatic process through which damaged cellular components and organelles are recycled and degraded during conditions of cellular stress [9]. The detailed mechanisms of autophagy have been described in numerous reports and review articles [10–13] and in this review, we briefly mention the general features of macroautophagy (Figure 1). The canonical autophagy pathway includes three different types of autophagy processes based on how the cargo is targeted and delivered to the lysosomes: macroautophagy, microautophagy, and chaperone-mediated autophagy. Among them, macroautophagy (usually referred to as autophagy) is triggered by numerous stress signals including starvation, hypoxia, damage to intracellular organelles, and microbial infection. In the initiation stage, the early phagophores (isolation membrane) are formed from the endoplasmic reticulum–mitochondria contact sites [14,15]. Numerous autophagy -related genes (ATGs) are involved in each step of the autophagy pathway. The ATG1/ULK complex and class III PI3K complex play essential roles in the formation of autophagosomes during the initiation stage [16,17]. The phagophores expand around the intracytoplasmic cargo and eventually grow into double membrane autophagosomes. The elongation and ultimate closure of autophagosomes are mediated through core machinery (i.e., a group of ubiquitin-like protein conjugation systems) such as the ATG5-ATG12-ATG16L1 complex and the ATG8 family, which is composed of microtubule-associated light chain 3 (LC3) [16,17]. Next, autophagosomes mature into autolysosomes by fusing with endosomes/lysosomes for the degradation of cytoplasmic cargo. This step depends on several non-ATG components including the endosomal sorting complex required for transport, soluble N-ethylmaleimide-sensitive factor attachment protein receptors, and RAB proteins [18,19].

## Overview of autophagy during mycobacterial infection

Mtb has developed several strategies to counteract autophagy, a well-known cellular response to various stresses including infections [20]. For example, Mtb Eis inhibits macrophage autophagy and cell death via a reactive oxygen species (ROS)-dependent pathway [21]. In addition, 6-kDa early secretory antigen target (ESAT6), a major ESX-1-mediated secretory protein, plays a role in the suppression of late-stage autophagy in human dendritic cells [22]. Since autophagy has emerged as a crucial protective process to restrict Mtb growth in host cells [23–25], it is worthwhile to focus on the function of autophagy against mycobacterial infection.

**Figure 2.**
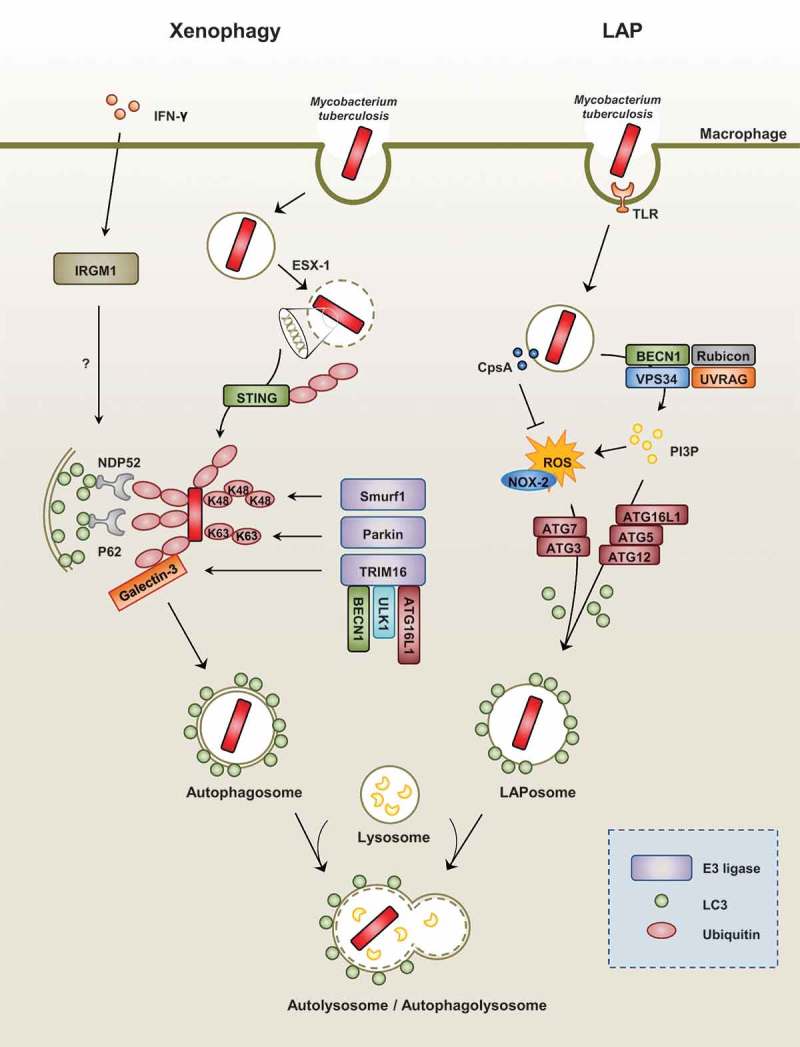
Xenophagy versus LAP during Mtb infection. (Left) During mycobacterial infection with Mtb, xenophagy is triggered by the cytoplasmic release of bacteria via its ESX-1 system. Next, the recognition of extracellular bacterial DNA by the STING-dependent pathway allows for the ubiquitination of bacteria. In addition, ubiquitin ligases such as Parkin and Smurf1 allow for the ubiquitination of different chain linkages (K63 and K48, respectively). TRIM16 cooperates with Galectin-3, ATG16L1, ULK1, and BECN1 for the subsequent ubiquitination of bacteria and autophagy activation. These ubiquitin chains recruit autophagy adaptors such as NDP52 and p62, which link to LC3 of the autophagosomal membranes. The exact function of IFN-γ-dependent IRGM1 (LRG47) in the regulation of selective autophagy requires further clarification. (Right) LAP is an LC3-conjugation process onto the single-membrane phagosome (LAPosome). It is triggered by pathogenic microbes, such as Mtb, through numerous receptors, including toll-like receptor signals. During the LAP process, recruitment of the class III PI3-kinase complex (composed of VPS34, Beclin-1, UVRAG, and Rubicon) increases the production of phosphatidylinositol-3-phosphate (PI3P), which is needed to stabilize the NOX2 complex for the production of reactive oxygen species and the recruitment of autophagic proteins (ATG5, ATG12, ATG16L, ATG7, and ATG3) for the conjugation of lipidated LC3-II to the LAPosomal membrane. Mtb protein CpsA is required for bacteria to block NADPH oxidase activity in order to evade killing by LAP.

Traditionally, canonical autophagy activation is considered nonspecific, and is involved in cell survival and homeostasis. Noncanonical autophagy includes selective autophagy, which targets a variety of components, macromolecules, and intracellular microbes, as well as LC3-associated phagocytosis (LAP) [26]. When the autophagic machinery targets intracellular pathogens, it is called “xenophagy,” a form of selective autophagy. Both the canonical and noncanonical autophagy pathways play pivotal roles in host defense and innate immune responses against intracellular pathogens [2,9]. There are numerous elegant reviews for the mechanism, function, and regulation of canonical autophagy [12,27,28]. Thus we here discuss a brief summary of xenophagy and LAP in terms of mycobacterial infection (Figure 2).

### Selective autophagy (xenophagy) targeting mycobacteria

During mycobacterial infection, xenophagy against Mtb infection is triggered by the cytoplasmic release of bacteria through its ESX-1 system. The STING-dependent cytosolic pathway and autophagic receptors p62 and NDP52 play a critical role in the xenophagic elimination of Mtb [29]. The subsequent ubiquitination and host defense against Mtb are dependent on the ubiquitin ligase Parkin [30]. The ubiquitin ligase Smurf1, which functions primarily in K48-linked ubiquitination, was recently found to play an essential role in the activation of selective autophagy, host defense against Mtb, and lung inflammation [31]. In addition, TRIM16, in cooperation with Galectin-3 and ATG16L1, is required for ubiquitination and autophagy in response to lysosomal damage, and contributes to autophagic protection against Mtb infection [32].

Earlier studies have demonstrated the importance of murine immunity-related p47 guanosine triphosphatase family M protein 1 (IRGM1) (also known as LRG47) in IFN-γ-dependent host defense against several intracellular infections, including mycobacterial infection [33]. In addition, IRGM1 and human IRGM (the human homolog of mouse *Irgm/Lrg47*) have roles in the autophagic elimination of mycobacteria [34,35] and contribute genetically to Crohn’s disease and TB [25,36,37]. Recent studies have revealed the molecular mechanisms by which IRGM functions in the regulation of autophagy and the promotion of antimicrobial effects. IRGM works by stabilizing AMP-activated protein kinase (AMPK) and maintaining and associating with autophagy factors ULK1, ATG14, and ATG16L1 [38]. However, the exact contribution of IRGM to the regulation of selective autophagy during mycobacterial infection requires further clarification. In addition, the IRGM/murine ortholog IRGM1 may play multiple regulatory functions in addition to autophagy in terms of infection and inflammation [39].

Furthermore, a recent study by Kimmey et al. showed that multiple autophagy-related genes did not contribute to host resistance to Mtb infection in mouse models in vivo [40]. Importantly, the authors could not rule out that autophagy restricts the mycobacterial growth [40]. Future studies are warranted which pathways/factors are critically required for antimicrobial responses by autophagy activation in human cells as well as in mouse models.

### LAP and mycobacterial infection

LAP is an LC3 conjugation process onto the phagosomal membrane during the phagocytosis of pathogenic microbes or apoptotic cells [41–44]. As a noncanonical autophagy pathway, LAP does not lead to the formation of double membrane structures [45,46]. Upon Toll-like receptor (TLR) signaling, the autophagic proteins LC3 and Beclin-1 are directly recruited to the single-membrane phagosome and promote phagosomal maturation and intracellular bacterial killing [45]. Indeed, LAP is triggered by the recognition of pathogenic microbes or dead cells through numerous receptors including Fc-receptors, TLRs, the C-type lectin receptor Dectin-1, or the phosphatidylserine receptor T cell immunoglobulin mucin-4 [41,47,48]. LAP does not depend on the activity of the core pre-initiation complex of conventional autophagy machinery but rather involves the function of certain autophagic proteins including BECN1, ATG5, ATG7, and ATG3 [49,50]. Importantly, RUBCN/Rubicon is a critical regulator of LAP and a distinguished molecule in the canonical autophagy pathway [51]. During the LAP process, the phagocytosed cargo within a single-membrane phagosome, called the LAPosome, is decorated with PI3P produced by the class III PI3-kinase complex (VPS15, VPS34, Beclin-1, UVRAG, and Rubicon). RUBCN and PI3P are required for the stabilization of CYBB/NOX-2 to generate ROS and the conjugation of lipidated LC3-II to the LAPosomal membrane to enhance phagosomal maturation [50,51]. Since Mtb activates numerous PRRs [52], LAP is being thought to be essential for the regulation of antimicrobial an inflammatory responses during mycobacterial infection. However, recent study showed that Mtb protein CpsA enabled bacteria to evade NADPH oxidase-dependent ROS signaling and LAP [53]. Still, it remains unclear what pathway is mainly involved (i.e., either LAP or xenophagy or both) upon Mtb infection from the perspective of host defense.

## Potential anti-TB reagents that target autophagy

As mentioned above, HDT is an emerging concept for emerging and re-emerging infectious diseases including TB via the enhancement of protective immunity [5]. Here we discuss the potential drug candidates that target autophagy for the development of HDT-TB. For clear understanding, we also summarized drug types and their mechanisms in Table 1 [54–76].

**Table 1.** Potential adjunctive anti-TB therapeutics targeting autophagy.

Candidates	Mechanisms	References
**Vitamin D receptor signaling**		
1,25-dihydroxyvitamin D3	Induction of cathelicidin, autophagy gene transcription IL1R signaling and DEFB4/HBD2 induction	[54, 57]
Mycobacterial LpqH antigen	Functional VDR activation through TLR2/1/CD14-Ca^2+^-AMPK-p38 MAPK signaling	[55]
4-phenylbutyrate	LL-37 expression, P2RX7 receptor signaling, intracellular calcium influx and AMPK pathways	[56]
IFN-γ	Crosstalk with vitamin D-dependent pathway	[58,59]
**Nuclear receptors and agonists**		
NR1D1	Increase of MAPL1LC3-II and LAMP1	[60]
PPAR-α	Upregulation of autophagy and lysosomal genes	[61]
ESRRA	Transcriptional and post-tranaslational regulation of autophagy genes/protein	[62]
**AMPK pathway**		
Calcium-mobilizing agents	Ca(2+)/calmodulin-dependent kinase kinase-β (CaMKKβ)-mediated AMPK activation	[63]
AICAR	AMPK-PGC1α-mediated autophagy-related gene transcription	[64]
Cyclic peptides Ohmyungsamycins A/B	AMPK-mediated autophagy activation and regulation of inflammation	[65]
Phytochemicals; steroid glycoside chemicals	AMPK-ULK1-dependent pathway	[66]
Resveratrol	Activation of AMPK and sirtuin 1	[67,68]
**Small molecules/Chemicals**		
Gefitinib	Inhibition of p38 MAPK pathway; activation of autophagy	[69]
Fluoxetine	Increased secretion of TNF-α; induction of autophagy	[69]
Baicalin	Modulation of PI3K/Akt/mTOR pathway	[70]
Loperamide, Verapamil	Induction of autophagy; mechanisms are unknown	[71]
Src kinase inhibitors	Activation of autophagy; mechanisms are unknown	[72]
Carbamazepine	Inositol triphosphate (IP_3_) depletion; mTOR-independent mechanisms	[73]
**Antibiotics**		
Isoniazid, Pyrazinamide	Bacterial hydroxyl radicals and cellular ROS-dependent mechanisms	[74]
Thiostrepton	ER stress-mediated autophagy activation	[75]
Ambroxol	Induction of autophagy; mechanisms are unknown	[76]

### Vitamin D3 and VDR signaling

The impact of vitamin D and vitamin D receptor (VDR) signaling has been most extensively studied in the context of autophagy and host defense. Innate immune activation and VDR signaling are tightly linked in innate immune cells to converge into antimicrobial host defenses against Mtb infection [77]. Liu et al. [78] demonstrated that, in human monocytes/macrophages, activation of VDR signaling leads to the induction of cathelicidin, a cationic antimicrobial protein with killing effects against Mtb. Further work by Yuk et al. [54] showed that 1,25-D3 enhances the activation of antibacterial autophagy and the elimination of intracellular Mtb in human monocytes/macrophages via cathelicidin induction and autophagy gene activation. Interestingly, TLR signaling activation during Mtb infection triggers a complicated intracellular signaling pathway that induces the expression of the Cyp27b1 gene (1α-hydroxylase) in human monocytes/macrophages, thereby activating functional VDR signaling to amplify cathelicidin induction and antimicrobial responses [55,79]. Furthermore, 4-phenylbutyrate (PBA), given alone or in combination with vitamin D3, overcomes the Mtb-induced inhibition of cathelicidin LL-37 expression and enhances anti-mycobacterial effects [56,80]. Rekha [56] found that PBA-induced autophagy activation is mediated through numerous intracellular signaling pathways including LL-37 expression, P2RX7 receptor signaling, and the intracellular calcium and AMPK pathways. Importantly, Fabri et al. [59] showed that supplementation of vitamin D-deficient serum with 25-hydroxyvitamin D3 recovered IFN-γ-mediated autophagy and phagosome-lysosome fusion in human macrophages, which suggests the importance of sufficient levels of vitamin D for the maintenance of protective immunity against Mtb infection. However, there are conflicting results regarding the therapeutic effects of vitamin D3, presumably due to the variation in individual levels of basal vitamin D, treatment dose and duration, and the clinical stages of disease [3]. Further studies with proper clinical settings will clarify our understanding of the beneficial effects of vitamin D in terms of protection and therapeutics against TB.

### Nuclear receptors and agonists

Several nuclear receptor agonists have promising effects on host autophagy and antimicrobial defense in mycobacterial infection. Chandra et al. [60] showed that nuclear receptor subfamily 1, group D, member 1 (NRD1), an adopted orphan nuclear receptor, is involved in the enhancement of antimycobacterial effects and autophagy in human macrophages. NR1D1 contributes to the activation of autophagy and lysosomal biogenesis via an increase in MAP1LC3-II and the lysosomal protein LAMP1.

In two recent studies, our group also examined autophagy and the antimycobacterial effects of orphan nuclear receptors. First, activation of the adopted orphan nuclear receptor peroxisome proliferator-activated receptor α (PPARα) led to the upregulated colocalization of Mtb phagosomes and autophagosomes, as well as lysosomal biogenesis and lipid catabolism, thereby enhancing antimycobacterial effects in macrophages. PPARα agonists have positive transcriptional regulatory effects on numerous autophagy and lysosomal genes, particularly transcription factor EB (TFEB), which is critically involved in antimicrobial effects and controlling macrophage inflammatory responses during mycobacterial infection [61]. In addition, the orphan nuclear receptor estrogen-related receptor α (ERRα), a critical regulator of metabolic homeostasis, plays an essential role in the activation of autophagy and increased anti-mycobacterial responses via transcriptional and post-translational modifications of essential autophagy genes/proteins. Through a positive feed-forward loop between ERRα and SIRT1, ERRα overexpression promotes the deacetylation of several autophagy-related proteins including ATG5, BECN1, and ATG7. Either ERRα or SIRT1 activation contributes to the enhancement of phagosomal acidification and antimicrobial responses against Mtb infection, thus demonstrating the critical role of ERRα in the innate host defense during mycobacterial infection [62]. It is interesting to note that all of the nuclear receptors described above (NR1D1, PPARα, and ERRα) have crucial functions in various biological processes, particularly energy metabolism. Thus, future studies are required to understand the ultimate roles of various nuclear receptors in terms of host susceptibilities or defense during infection in the context of metabolic diseases.

### Targeting the AMPK and SIRT1 pathways

AMPK, a key metabolic and energy regulator that functions by sensing the AMP/ATP ratio, plays essential roles in the coordination of anabolic and catabolic activity via the phosphorylation of multiple proteins [81]. Numerous studies have shown the positive action of AMPK signaling in autophagy activation. Activated AMPK promotes autophagy by inhibiting mTOR, a suppressor of autophagy induction, or by activating ULK1/2, autophagy initiators [82,83]. Mtb infection of macrophages leads to robust activation of mTOR signaling but only a slight increase in AMPKα phosphorylation [64]. Indeed, the AMPK pathway enhances antibacterial autophagy induced by numerous reagents or drugs [24,84]. A variety of calcium-mobilizing agents activate autophagy through the AMPK pathway via Ca (2+)/calmodulin-dependent kinase kinase-β signaling [63]. In human monocytes/macrophages, vitamin D signaling activates autophagy and antimicrobial defense against Mtb infection via AMPK activation [55,56]. In addition, the AMPK-PPARγ coactivator-1 α (PGC1α) pathway plays a critical role in the promotion of autophagy-related gene transcription to enhance antibacterial autophagy and phagosomal acidification in macrophages during mycobacterial infection. Importantly, AMPK-PGC1α signaling contributes to increased mitochondrial biogenesis and respiratory function in Mtb-infected macrophages, suggestive of mitochondrial support by the same pathway that triggers autophagy during infection [64]. In addition, newly identified cyclic peptides ohmyungsamycins A and B promote phagosomal maturation and antimicrobial responses against Mtb infections by activating the AMPK pathway [65]. Recent studies that have used analytical chemistry approaches have identified several natural chemicals that enhance autophagy via an AMPK-ULK1-dependent pathway and that play roles in cognitive behaviors in mouse models of Alzheimer’s disease [66]. The SIRT1 activator resveratrol (RSV) triggers the production of intracytoplasmic calcium and activates AMPK to promote the autophagy-dependent lysosomal degradation of amyloid-β [67]. We recently showed that RSV facilitates the colocalization of mycobacterial phagosomes with autophagosomes, as well as the antimicrobial effects against Mtb in macrophages [65]. Vingtdeux [68] screened a chemical library with structural similarities to RSV and identified potent autophagy-activating agents that facilitate the activation of AMPK but inhibit the mTOR pathway. Although the drugs mentioned above showed promising activity in amyloid-β clearance, whether these drugs enhance the autophagic clearance of intracellular Mtb remains unknown.

### Autophagy-targeting small molecules

A variety of small molecules of natural and synthetic origin may be promising targets for HDT-TB by targeting autophagy. Stanley [69], using a small molecule-based chemical biological approach, showed that gefitinib (Iressa, ZD-1839), which targets epidermal growth factor receptor, effectively inhibits the replication of Mtb in macrophages and *in vivo*, inhibits phosphorylation of the p38 MAPK pathway, and induces autophagy to enhance intracellular Mtb clearance. In that study, fluoxetine, a selective serotonin reuptake inhibitor, also exhibited restrictive activity against intracellular mycobacteria and induced autophagy, presumably via the increased secretion of the pro-inflammatory cytokine tumor necrosis factor alpha in Mtb-infected macrophages. Baicalin, an herbal medicine, induces autophagy via the PI3K/Akt/mTOR pathway, and exhibits antimicrobial and anti-inflammatory effects against Mtb infection, which suggests that it may be a new candidate for improved TB treatment [70]. Furthermore, a recent study that evaluated potential anti-mycobacterial agents, including loperamide and verapamil, showed that these drugs induce autophagy, which is accompanied by significant inhibition of intracellular Mtb survival in murine alveolar macrophages [71]. In our earlier study [74], we found that the standard anti-TB drugs isoniazid and pyrazinamide promoted autophagy activation, which was required for effective therapeutic responses in macrophages and fly models during mycobacterial infection. A very recent paper from Choi et al. [76] also demonstrated that the lead compound ambroxol induced autophagy *in vitro* and *in vivo*, promoting mycobacterial killing, in murine macrophages. Interestingly, ambroxol showed potentiating rifampin activity in a murine model of Mtb infection.

Several studies have proven the effectiveness of HDT-TB against drug-resistant strains. A recent study showed that inhibiting Src kinase activity significantly suppresses the intracellular survival of Mtb H37Rv, particularly the MDR and XDR strains of Mtb [72]. Importantly, in the same study, Src inhibition was effective for infection of the XDR-Mtb strain in a guinea pig models, which suggests that it may also be a promising agent for HDT-TB therapeutics. Importantly, persistent activation of Src kinase results in the activation of mTOR, a well-known autophagy repressor, whereas the inhibition of Src kinase leads to a partial rescue of defective autophagy [85]. A study that screened U.S. Food and Drug Administration-approved drugs showed that the anti-convulsant drug carbamazepine leads to the induction of autophagy via inositol triphosphate depletion and AMPK activation [73]. In that same study, the drug also significantly inhibited highly virulent MDR-TB in the lungs of infected mice. These findings shed light on potential therapeutic strategies using drug repositioning in the field of HDT-TB.

## Challenges and perspectives of autophagy-adjunctive therapeutics

Autophagy-adjunctive therapeutics is an emerging concept for the improved treatment of TB. Although it is a promising strategy, even for controlling drug-resistant strains, according to *in vitro* and preclinical studies [72,73,86], current data on its clinical use are insufficient, and more evidence is need in future studies.

### Challenges of autophagy-adjunctive therapeutics for clinical use

In Table 2 [87–96], we summarized recent clinical studies on vitamin D and autophagy-adjunctive therapy for patients with pulmonary TB. As previously discussed, vitamin D treatment has diverse biological effects on host defense in TB including autophagy activation. Mily et al. [95] demonstrated that adjunctive therapy with vitamin D alone or in combination with PBA to standard short-course therapy enhanced clinical recovery, thus representing a possibility for HDT-TB. Salahuddin et al. [91] revealed that supplementation with high doses of vitamin D3 accelerated clinical and radiological improvements in all TB patients and increased host immune activation in patients with deficient baseline serum levels of vitamin D. Despite these promising results, vitamin D-based therapy remains controversial. Other studies [87,88] have shown that vitamin D3 supplementation does not reduce the time period of sputum culture conversion. In addition, high-dose vitamin D3 regimens safely correct vitamin D3 deficiency without improving the rate of sputum Mtb clearance [89]. Inconsistent results regarding vitamin D-based HDT-TB may be due to the basal levels of vitamin D in the patient population, VDR polymorphisms, and geographic and/or ethnic variation associated with TB susceptibility [78,97,98]. In addition, it is difficult to show the comparative effects of randomized clinical trials in which standard TB drugs are given together with the adjuvant drug (e.g., vitamin D) or placebo. In fact, this issue may explain the null effect on the primary endpoint (sputum-conversion) found in many studies. Future studies on personalized medicine should be conducted to clarify the effective clinical use of vitamin D as a supplement for TB, MDR-TB, and latent TB infection. At present, few clinical trials have examined potential treatments for TB other than vitamin D. Trials of repositioning drugs for combating TB are ongoing. Repositioned drugs such as gefitinib, fluoxetine, loperamide, and verapamil could represent promising candidates and should be validated in well-designed clinical studies as adjuvant therapeutics for TB.

**Table 2.** Clinical studies of autophagy-adjunctive therapeutics for tuberculosis.

Study name		Phase	Results	Clinical trials. gov Identifier*	References
**Vitamin D replacement**					
Trial of Adjunctive Vitamin D in TB Treatment	III		Vitamin D did not significantly affect time to sputum culture conversion	NCT 419068	[87]
A Clinical Trial to Study the Effect of Vitamin D to Treatment in New Pulmonary TB Patients	II		Vitamin D supplementation did not reduce time to sputum culture conversion	NCT 366470	[88]
Impact of Vitamin D supplementation on Host Immunity to Mtb and Response to Treatment	II		A high-dose vitamin D3 regimen safely corrected vitamin D deficiency but did not improve the rate of sputum Mtb clearance over 16 weeks in this pulmonary TB cohort	NCT 918086	[89]
Replacement of Vitamin D in Patients With Active TB	II		High doses of vitamin D accelerated clinical, radiographic improvement in all TB patients and increased immune activation in patients with deficient serum vitamin D levels	NCT 1130311	[90,91]
Role of Vitamin D in Innate Immunity to TB	II		Vitamin D supplementation for 6 months had significant favorable effects on serum 25(OH) D concentrations and on growth in stature	NCT 1244204	[92]
Effects of Vitamin D Supplementation on Antimycobacterial Immunity	II		A single oral dose of 2.5 mg vitamin D significantly enhanced the ability of participants' whole blood to restrict BCG-lux luminescence *in vitro*	NCT 00157066	[93]
**Vitamin D + other replacement**					
L-arginine and Vitamin D Adjunctive Therapy in Pulmonary TB	III		Neither vitamin D nor L-arginine supplementation affected TB outcomes	NCT 677339	[94]
Clinical Trial of PBA and Vitamin D in TB	II		Adjunct therapy with PBA+vitamin D3 or vitamin D3 or PBA to standard short-course therapy demonstrated beneficial effects towards clinical recovery	NCT 01580007	[95]
**Drug repositioning**					
HDT-TB (Everolimus, Auranofin)	II		Enrolling	NCT 2968927	
Doxycycline in human pulmonary TB	II		Enroll: completed	NCT 2774993	[96]

BCG, Bacille Calmette-Guérin * Further details for trial with NCT numbers can be accessed at http://clinicaltrials.gov.

In addition, there are several issues associated with the failure of TB treatment due to drug resistance, poor compliance, and severe side effects. Therefore, the enrollment of carefully selected and appropriate patients for specific drug regimens may be one of the most important factors when evaluating autophagy-adjunctive therapeutics as new and improved therapeutic options for refractory TB. In addition, there is a lack of studies on drug delivery and treatment duration in autophagy-adjunctive therapy combined with conventional antibiotics treatment. It is necessary to deliver the drug to the affected tissues/organs to avoid nontarget cell exposure and unwanted side effects [8].

### Limitation and perspectives of autophagy-adjunctive therapeutics

A major limitation of this review is the limited information obtained from the in vitro studies demonstrating that autophagy activation is beneficial for inhibiting Mtb survival in host cells. In addition, there might be off-target effects by autophagy-modulating small molecules or agents. Studies using gene-manipulated mouse models of autophagy have provided essential knowledge for understanding how autophagy functions against mycobacterial infection. There is evidence showing the beneficial effects of GTPase IRGM on cell-autonomous protective immunity against mycobacterial infection in murine and human cells [34,35]. However, there is clear evidence of species-specific differences. For example, the major axis of autophagy and host defense involving vitamin D and cathelicidin is not functional in mouse models [99]. Future studies of autophagy-targeted anti-mycobacterial responses in vivo are warranted for the development of new treatments against TB and the assessment of off-target effects as well as species-specific similarities and differences.

It would also be interesting to identify the actual effector arms in autophagy-mediated killing of bacteria. Ponpuak et al. [100] described the existence of a novel antimicrobial peptide that can directly kill Mtb. They successfully demonstrated that the autophagic adaptor protein p62 is involved in the transport of rpS30 peptide to the mycobacterial phagosome and in the autophagy prevention of tubercle bacilli. However, the degree to which rpS30 or cathelicidin is actually involved in bacterial killing in autophagy-mediated HDTs is still unknown. We also believe that an acidic environment in phagolysosomal acidification can directly damage live Mtb [101] and that numerous lysosomal enzymes are involved in such circumstances. Thus, future studies are needed to investigate this aspect further, and it would be promising to identify new antimicrobial peptides that directly target Mtb to advance autophagy-based HDT studies.

## Concluding remarks

During recent years, numerous advances have been made in our understanding of the discovery and functional characterization of autophagy-based antimicrobial agents and pathways. Despite the potential advantages of autophagy-activating agents for HDT against TB, many challenges remain with regard to the detailed molecular mechanisms by which the activation of autophagy enhances the limitation of Mtb replication in host cells. There is a possibility that the different pathways/mechanisms involved may participate in the enhancement of autophagic clearance of intracellular Mtb depending on diverse autophagy-activating agents. The known HDT drugs play important roles in pathologic inflammation, phagolysosomal fusion, lysosomal functions, and the antimicrobial activity of host cells infected with Mtb infection. Although autophagy-activating agents may act as therapeutic candidates for HDT-TB, it is possible that many other biological pathways, in addition to autophagy, contribute to the host defense against TB infection. For example, the most widely used reagent for HDT-TB is vitamin D, a therapeutic reagent for TB used before the antibiotic era [102]. In addition to its autophagy-activating ability, vitamin D has pleiotropic effects on infected cells and/or tissues to promote direct antimicrobial defense via cathelicidin and by regulating inflammation. Another challenge in the field relates to clinical applications, namely, whether drug administration could be targeted to disease sites or whether autophagy-adjunctive therapies could reduce the duration of antibiotic treatment. Continuous and future studies on autophagy-based HDT therapeutic candidates will not only provide insight into the antibacterial function of autophagy, but may also contribute to potential therapeutic improvements against TB.

## References

[1] Pareja ME, Colombo MI. Autophagic clearance of bacterial pathogens: molecular recognition of intracellular microorganisms. Front Cell Infect Microbiol. 2013;3:54. 24137567 10.3389/fcimb.2013.00054 PMC3786225

[2] Bah A, Vergne I Macrophage autophagy and bacterial infections. Front Immunol. 2017;8:1483. 29163544 10.3389/fimmu.2017.01483 PMC5681717

[3] Kolloli A, Subbian S Host-directed therapeutic strategies for tuberculosis. Front Med (Lausanne). 2017;4:171. 29094039 10.3389/fmed.2017.00171 PMC5651239

[4] Kimmey JM, Stallings CL Bacterial pathogens versus autophagy: implications for therapeutic interventions. Trends Mol Med. 2016 12;22(12):1060–1076. 27866924 10.1016/j.molmed.2016.10.008 PMC5215815

[5] Kaufmann SHE, Dorhoi A, Hotchkiss RS, et al Host-directed therapies for bacterial and viral infections. Nat Rev Drug Discov. 2018 1;17(1):35–56. 28935918 10.1038/nrd.2017.162 PMC7097079

[6] Zumla A, Maeurer M Host-Directed therapies for tackling multi-drug resistant tuberculosis: learning from the Pasteur-Bechamp debates. Clin Infect Dis. 2015 11 1;61(9):1432–1438. 26219693 10.1093/cid/civ631

[7] Zumla A, Rao M, Dodoo E, et al Potential of immunomodulatory agents as adjunct host-directed therapies for multidrug-resistant tuberculosis. BMC Med. 2016 6;15(14):89. 10.1186/s12916-016-0635-1 PMC4908783 27301245

[8] Gupta A, Misra A, Deretic V Targeted pulmonary delivery of inducers of host macrophage autophagy as a potential host-directed chemotherapy of tuberculosis. Adv Drug Deliv Rev. 2016 7;1(102):10–20. 10.1016/j.addr.2016.01.016 PMC5285306 26829287

[9] Y GL M, Zitvogel L, Kroemer G Autophagy and cellular immune responses. Immunity. 2013;39:211–227. 23973220 10.1016/j.immuni.2013.07.017

[10] Klionsky DJ, Emr SD Autophagy as a regulated pathway of cellular degradation. Science. 2000 12 1;290(5497):1717–1721. 11099404 10.1126/science.290.5497.1717 PMC2732363

[11] Mizushima N, Levine B, Cuervo AM, et al Autophagy fights disease through cellular self-digestion. Nature. 2008 2 28;451(7182):1069–1075. 18305538 10.1038/nature06639 PMC2670399

[12] Levine B, Kroemer G Autophagy in the pathogenesis of disease. Cell. 2008 1 11;132(1):27–42. 18191218 10.1016/j.cell.2007.12.018 PMC2696814

[13] Codogno P, Meijer AJ Autophagy and signaling: their role in cell survival and cell death. Cell Death Differ. 2005 11;12(Suppl 2):1509–1518. 16247498 10.1038/sj.cdd.4401751

[14] Axe EL, Walker SA, Manifava M, et al Autophagosome formation from membrane compartments enriched in phosphatidylinositol 3-phosphate and dynamically connected to the endoplasmic reticulum. J Cell Biol. 2008 8 25;182(4):685–701. 18725538 10.1083/jcb.200803137 PMC2518708

[15] Hamasaki M, Furuta N, Matsuda A, et al Autophagosomes form at ER-mitochondria contact sites. Nature. 2013 3 21;495(7441):389–393. 23455425 10.1038/nature11910

[16] Feng Y, He D, Yao Z, et al The machinery of macroautophagy. Cell Res. 2014 1;24(1):24–41. 24366339 10.1038/cr.2013.168 PMC3879710

[17] Shibutani ST, Yoshimori T A current perspective of autophagosome biogenesis. Cell Res. 2014 1;24(1):58–68. 24296784 10.1038/cr.2013.159 PMC3879706

[18] Lata S, Schoehn G, Solomons J, et al Structure and function of ESCRT-III. Biochem Soc Trans. 2009 2;37(Pt(1)):156–160. 19143622 10.1042/BST0370156

[19] Furuta N, Yoshimori T, Amano A Mediatory molecules that fuse autophagosomes and lysosomes. Autophagy. 2010 4;6(3):417–418. 20400858 10.4161/auto.6.3.11418

[20] de Chastellier C The many niches and strategies used by pathogenic mycobacteria for survival within host macrophages. Immunobiology. 2009;214(7):526–542. 19261352 10.1016/j.imbio.2008.12.005

[21] Shin DM, Jeon BY, Lee HM, et al *Mycobacterium tuberculosis* eis regulates autophagy, inflammation, and cell death through redox-dependent signaling. PLoS Pathog. 2010 12 16;6(12):e1001230. 21187903 10.1371/journal.ppat.1001230 PMC3002989

[22] Romagnoli A, Etna MP, Giacomini E, et al ESX-1 dependent impairment of autophagic flux by *Mycobacterium tuberculosis* in human dendritic cells. Autophagy. 2012 9;8(9):1357–1370. 22885411 10.4161/auto.20881 PMC3442882

[23] Levine B, Deretic V Unveiling the roles of autophagy in innate and adaptive immunity. Nat Rev Immunol. 2007 10;7(10):767–777. 17767194 10.1038/nri2161 PMC7097190

[24] Fabri M, Realegeno SE, Jo EK, et al Role of autophagy in the host response to microbial infection and potential for therapy. Curr Opin Immunol. 2011 2;23(1):65–70. 21071195 10.1016/j.coi.2010.10.010 PMC3042547

[25] Songane M, Kleinnijenhuis J, Netea MG, et al The role of autophagy in host defence against *Mycobacterium tuberculosis* infection. Tuberculosis (Edinb). 2012 9;92(5):388–396. 22683183 10.1016/j.tube.2012.05.004

[26] Sil P, Muse G, Martinez J A ravenous defense: canonical and non-canonical autophagy in immunity. Curr Opin Immunol. 2017 11;7(50):21–31. 10.1016/j.coi.2017.10.004 PMC5857463 29125936

[27] Mizushima N Autophagy: process and function. Genes Dev. 2007 11 15;21(22):2861–2873. 18006683 10.1101/gad.1599207

[28] Glick D, Barth S, Macleod KF Autophagy: cellular and molecular mechanisms. J Pathol. 2010 5;221(1):3–12. 20225336 10.1002/path.2697 PMC2990190

[29] Watson RO, Manzanillo PS, Cox JS Extracellular *M. tuberculosis* DNA targets bacteria for autophagy by activating the host DNA-sensing pathway. Cell. 2012 8 17;150(4):803–815. 22901810 10.1016/j.cell.2012.06.040 PMC3708656

[30] Manzanillo PS, Ayres JS, Watson RO, et al The ubiquitin ligase parkin mediates resistance to intracellular pathogens. Nature. 2013 9 26;501(7468):512–516. 24005326 10.1038/nature12566 PMC3886920

[31] Franco LH, Nair VR, Scharn CR, et al The ubiquitin ligase Smurf1 functions in selective autophagy of *Mycobacterium tuberculosis* and anti-tuberculous host defense. Cell Host Microbe. 2017 1 11;21(1):59–72. 28017659 10.1016/j.chom.2016.11.002 PMC5699477

[32] Chauhan S, Kumar S, Jain A, et al TRIMs and Galectins globally cooperate and TRIM16 and Galectin-3 co-direct autophagy in endomembrane damage homeostasis. Dev Cell. 2016 10 10;39(1):13–27. 27693506 10.1016/j.devcel.2016.08.003 PMC5104201

[33] Feng CG, Collazo-Custodio CM, Eckhaus M, et al Mice deficient in LRG-47 display increased susceptibility to mycobacterial infection associated with the induction of lymphopenia. J Immunol. 2004 1 15;172(2):1163–1168. 14707092 10.4049/jimmunol.172.2.1163

[34] Gutierrez MG, Master SS, Singh SB, et al Autophagy is a defense mechanism inhibiting BCG and *Mycobacterium tuberculosis* survival in infected macrophages. Cell. 2004 12 17;119(6):753–766. 15607973 10.1016/j.cell.2004.11.038

[35] Singh SB, Davis AS, Taylor GA, et al Human IRGM induces autophagy to eliminate intracellular mycobacteria. Science. 2006 9 8;313(5792):1438–1441. 16888103 10.1126/science.1129577

[36] The Wellcome Trust Case Control Consortium Genome-wide association study of CNVs in 16,000 cases of eight common diseases and 3,000 shared controls. Nature. 2010 4 1;464(7289):713–720. 20360734 10.1038/nature08979 PMC2892339

[37] The Wellcome Trust Case Control Consortium Genome-wide association study of 14,000 cases of seven common diseases and 3,000 shared controls. Nature. 2007 6 7;447(7145):661–678. 17554300 10.1038/nature05911 PMC2719288

[38] Chauhan S, Mandell MA, Deretic V IRGM governs the core autophagy machinery to conduct antimicrobial defense. Mol Cell. 2015 5 7;58(3):507–521. 25891078 10.1016/j.molcel.2015.03.020 PMC4427528

[39] Matsuzawa T, Kim BH, Shenoy AR, et al IFN-gamma elicits macrophage autophagy via the p38 MAPK signaling pathway. J Immunol. 2012 7 15;189(2):813–818. 22675202 10.4049/jimmunol.1102041 PMC3392356

[40] Kimmey JM, Huynh JP, Weiss LA, et al Unique role for ATG5 in neutrophil-mediated immunopathology during *M. tuberculosis* infection. Nature. 2015 12 24;528(7583):565–569. 26649827 10.1038/nature16451 PMC4842313

[41] Martinez J, Almendinger J, Oberst A, et al Microtubule-associated protein 1 light chain 3 alpha (LC3)-associated phagocytosis is required for the efficient clearance of dead cells. Proc Natl Acad Sci U S A. 2011 10 18;108(42):17396–17401. 21969579 10.1073/pnas.1113421108 PMC3198353

[42] Galluzzi L, Baehrecke EH, Ballabio A, et al Molecular definitions of autophagy and related processes. Embo J. 2017 7 3;36(13):1811–1836. 28596378 10.15252/embj.201796697 PMC5494474

[43] Mitchell G, Isberg RR Innate immunity to intracellular pathogens: balancing microbial elimination and inflammation. Cell Host Microbe. 2017 8 9;22(2):166–175. 28799902 10.1016/j.chom.2017.07.005 PMC5562164

[44] Panneerdoss S, Viswanadhapalli S, Abdelfattah N, et al Cross-talk between miR-471-5p and autophagy component proteins regulates LC3-associated phagocytosis (LAP) of apoptotic germ cells. Nat Commun. 2017 9 19;8(1):598. 28928467 10.1038/s41467-017-00590-9 PMC5605700

[45] Sanjuan MA, Dillon CP, Tait SW, et al Toll-like receptor signalling in macrophages links the autophagy pathway to phagocytosis. Nature. 2007 12 20;450(7173):1253–1257. 18097414 10.1038/nature06421

[46] Mehta P, Henault J, Kolbeck R, et al Noncanonical autophagy: one small step for LC3, one giant leap for immunity. Curr Opin Immunol. 2014;26:69–75. 24556403 10.1016/j.coi.2013.10.012

[47] Henault J, Martinez J, Riggs JM, et al Noncanonical autophagy is required for type I interferon secretion in response to DNA-immune complexes. Immunity. 2012 12 14;37(6):986–997. 23219390 10.1016/j.immuni.2012.09.014 PMC3786711

[48] Ma J, Becker C, Lowell CA, et al Dectin-1-triggered recruitment of light chain 3 protein to phagosomes facilitates major histocompatibility complex class II presentation of fungal-derived antigens. J Biol Chem. 2012 10 5;287(41):34149–34156. 22902620 10.1074/jbc.M112.382812 PMC3464523

[49] Bandyopadhyay U, Overholtzer M LAP: the protector against autoimmunity. Cell Res. 2016 8;26(8):865–866. 27297234 10.1038/cr.2016.70 PMC4973329

[50] Sprenkeler EG, Gresnigt MS van de Veerdonk FL. LC3-associated phagocytosis: a crucial mechanism for antifungal host defence against *Aspergillus fumigatus*. Cell Microbiol. 2016 9;18(9):1208–1216. 27185357 10.1111/cmi.12616

[51] Martinez J, Malireddi RK, Lu Q, et al Molecular characterization of LC3-associated phagocytosis reveals distinct roles for Rubicon, NOX2 and autophagy proteins. Nat Cell Biol. 2015 7;17(7):893–906. 26098576 10.1038/ncb3192 PMC4612372

[52] Ferwerda G, Girardin SE, Kullberg BJ, et al NOD2 and toll-like receptors are nonredundant recognition systems of *Mycobacterium tuberculosis*. PLoS Pathog. 2005 11;1(3):279–285. 16322770 10.1371/journal.ppat.0010034 PMC1291354

[53] Koster S, Upadhyay S, Chandra P, et al *Mycobacterium tuberculosis* is protected from NADPH oxidase and LC3-associated phagocytosis by the LCP protein CpsA. Proc Natl Acad Sci U S A. 2017 10 10;114(41):E8711–E8720. 28973896 10.1073/pnas.1707792114 PMC5642705

[54] Yuk JM, Shin DM, Lee HM, et al Vitamin D3 induces autophagy in human monocytes/macrophages via cathelicidin. Cell Host Microbe. 2009 9 17;6(3):231–243. 19748465 10.1016/j.chom.2009.08.004

[55] Shin DM, Yuk JM, Lee HM, et al Mycobacterial lipoprotein activates autophagy via TLR2/1/CD14 and a functional vitamin D receptor signalling. Cell Microbiol. 2010 11;12(11):1648–1665. 20560977 10.1111/j.1462-5822.2010.01497.x PMC2970753

[56] Rekha RS, Rao Muvva SS, Wan M, et al Phenylbutyrate induces LL-37-dependent autophagy and intracellular killing of *Mycobacterium tuberculosis* in human macrophages. Autophagy. 2015;11(9):1688–1699. 26218841 10.1080/15548627.2015.1075110 PMC4590658

[57] Verway M, Bouttier M, Wang TT, et al Vitamin D induces interleukin-1beta expression: paracrine macrophage epithelial signaling controls *M. tuberculosis* infection. PLoS Pathog. 2013;9(6):e1003407. 23762029 10.1371/journal.ppat.1003407 PMC3675149

[58] Edfeldt K, Liu PT, Chun R, et al T-cell cytokines differentially control human monocyte antimicrobial responses by regulating vitamin D metabolism. Proc Natl Acad Sci U S A. 2010 12 28;107(52):22593–22598. 21149724 10.1073/pnas.1011624108 PMC3012480

[59] Fabri M, Stenger S, Shin DM, et al Vitamin D is required for IFN-gamma-mediated antimicrobial activity of human macrophages. Sci Transl Med. 2011 10 12;3(104):104ra102. 10.1126/scitranslmed.3003045 PMC3269210 21998409

[60] Chandra V, Bhagyaraj E, Nanduri R, et al NR1D1 ameliorates *Mycobacterium tuberculosis* clearance through regulation of autophagy. Autophagy. 2015 11 2;11(11):1987–1997. 26390081 10.1080/15548627.2015.1091140 PMC4824569

[61] Kim YS, Lee HM, Kim JK, et al PPAR-alpha activation mediates innate host defense through induction of TFEB and lipid catabolism. J Immunol. 2017 4 15;198(8):3283–3295. 28275133 10.4049/jimmunol.1601920

[62] Kim SY, Yang CS, Lee HM, et al ESRRA (estrogen-related receptor alpha) is a key coordinator of transcriptional and post-translational activation of autophagy to promote innate host defense. Autophagy. 2018;14(1):152–168. 28841353 10.1080/15548627.2017.1339001 PMC5846564

[63] Hoyer-Hansen M, Bastholm L, Szyniarowski P, et al Control of macroautophagy by calcium, calmodulin-dependent kinase kinase-beta, and Bcl-2. Mol Cell. 2007 1 26;25(2):193–205. 17244528 10.1016/j.molcel.2006.12.009

[64] Yang CS, Kim JJ, Lee HM, et al The AMPK-PPARGC1A pathway is required for antimicrobial host defense through activation of autophagy. Autophagy. 2014 5;10(5):785–802. 24598403 10.4161/auto.28072 PMC5119058

[65] Kim TS, Shin YH, Lee HM, et al Ohmyungsamycins promote antimicrobial responses through autophagy activation via AMP-activated protein kinase pathway. Sci Rep. 2017 6 13;7(1):3431. 28611371 10.1038/s41598-017-03477-3 PMC5469788

[66] Fan Y, Wang N, Rocchi A, et al Identification of natural products with neuronal and metabolic benefits through autophagy induction. Autophagy. 2017 1 2;13(1):41–56. 27791467 10.1080/15548627.2016.1240855 PMC5240827

[67] Vingtdeux V, Giliberto L, Zhao H, et al AMP-activated protein kinase signaling activation by resveratrol modulates amyloid-beta peptide metabolism. J Biol Chem. 2010 3 19;285(12):9100–9113. 20080969 10.1074/jbc.M109.060061 PMC2838330

[68] Vingtdeux V, Chandakkar P, Zhao H, et al Novel synthetic small-molecule activators of AMPK as enhancers of autophagy and amyloid-beta peptide degradation. FASEB J. 2011 1;25(1):219–231. 20852062 10.1096/fj.10-167361 PMC3005419

[69] Stanley SA, Barczak AK, Silvis MR, et al Identification of host-targeted small molecules that restrict intracellular *Mycobacterium tuberculosis* growth. PLoS Pathog. 2014 2;10(2):e1003946. 24586159 10.1371/journal.ppat.1003946 PMC3930586

[70] Zhang Q, Sun J, Wang Y, et al Antimycobacterial and anti-inflammatory mechanisms of Baicalin via induced autophagy in macrophages Infected with *Mycobacterium tuberculosis*. Front Microbiol. 2017;8:2142. 29163427 10.3389/fmicb.2017.02142 PMC5673628

[71] Juarez E, Carranza C, Sanchez G, et al Loperamide restricts intracellular growth of *Mycobacterium tuberculosis* in lung macrophages. Am J Respir Cell Mol Biol. 2016 12;55(6):837–847. 27468130 10.1165/rcmb.2015-0383OC

[72] Chandra P, Rajmani RS, Verma G, et al Targeting drug-sensitive and -resistant strains of *Mycobacterium tuberculosis* by inhibition of Src family kinases lowers disease burden and pathology. mSphere. 2016 Mar-Apr;1(2):e00043-15. 10.1128/mSphere.00043-15 PMC4894694 27303736

[73] Schiebler M, Brown K, Hegyi K, et al Functional drug screening reveals anticonvulsants as enhancers of mTOR-independent autophagic killing of *Mycobacterium tuberculosis* through inositol depletion. EMBO Mol Med. 2015 2;7(2):127–139. 25535254 10.15252/emmm.201404137 PMC4328644

[74] Kim JJ, Lee HM, Shin DM, et al Host cell autophagy activated by antibiotics is required for their effective antimycobacterial drug action. Cell Host Microbe. 2012 5 17;11(5):457–468. 22607799 10.1016/j.chom.2012.03.008

[75] Zheng Q, Wang Q, Wang S, et al Thiopeptide antibiotics exhibit a dual mode of action against intracellular pathogens by affecting both host and microbe. Chem Biol. 2015 8 20;22(8):1002–1007. 26211364 10.1016/j.chembiol.2015.06.019

[76] Choi SW, Gu Y, Peters RS, et al Ambroxol induces autophagy and potentiates rifampin antimycobacterial activity. Antimicrob Agents Chemother. 2018 9;62(9):e01019-18. 10.1128/AAC.01019-18 PMC6125555 30012752

[77] Liu PT, Krutzik SR, Modlin RL Therapeutic implications of the TLR and VDR partnership. Trends Mol Med. 2007 3;13(3):117–124. 17276732 10.1016/j.molmed.2007.01.006

[78] Liu PT, Stenger S, Li H, et al Toll-like receptor triggering of a vitamin D-mediated human antimicrobial response. Science. 2006 3 24;311(5768):1770–1773. 16497887 10.1126/science.1123933

[79] Liu PT, Schenk M, Walker VP, et al Convergence of IL-1beta and VDR activation pathways in human TLR2/1-induced antimicrobial responses. PLoS One. 2009 6 5;4(6):e5810. 19503839 10.1371/journal.pone.0005810 PMC2686169

[80] Mily A, Rekha RS, Kamal SM, et al Oral intake of phenylbutyrate with or without vitamin D3 upregulates the cathelicidin LL-37 in human macrophages: a dose finding study for treatment of tuberculosis. BMC Pulm Med. 2013 4 16;13:23. 23590701 10.1186/1471-2466-13-23 PMC3637063

[81] Hardie DG, Ross FA, Hawley SA AMPK: a nutrient and energy sensor that maintains energy homeostasis. Nat Rev Mol Cell Biol. 2012 3 22;13(4):251–262. 22436748 10.1038/nrm3311 PMC5726489

[82] Kim J, Kundu M, Viollet B, et al AMPK and mTOR regulate autophagy through direct phosphorylation of Ulk1. Nat Cell Biol. 2011 2;13(2):132–141. 21258367 10.1038/ncb2152 PMC3987946

[83] Alers S, Loffler AS, Wesselborg S, et al Role of AMPK-mTOR-Ulk1/2 in the regulation of autophagy: cross talk, shortcuts, and feedbacks. Mol Cell Biol. 2012 1;32(1):2–11. 22025673 10.1128/MCB.06159-11 PMC3255710

[84] Hoyer-Hansen M, Jaattela M AMP-activated protein kinase: a universal regulator of autophagy? Autophagy. 2007 Jul-Aug;3(4):381–383. 17457036 10.4161/auto.4240

[85] Pal R, Palmieri M, Loehr JA, et al Src-dependent impairment of autophagy by oxidative stress in a mouse model of Duchenne muscular dystrophy. Nat Commun. 2014 7;16(5):4425. 10.1038/ncomms5425 PMC4101811 25028121

[86] Kidwai S, Park CY, Mawatwal S, et al Dual mechanism of action of 5-nitro-1,10-phenanthroline against *Mycobacterium tuberculosis*. Antimicrob Agents Chemother. 2017 11;61(11):e00969-17. 10.1128/AAC.00969-17 PMC5655107 28893784

[87] Martineau AR, Timms PM, Bothamley GH, et al High-dose vitamin D(3) during intensive-phase antimicrobial treatment of pulmonary tuberculosis: a double-blind randomised controlled trial. Lancet. 2011 1 15;377(9761):242–250. 21215445 10.1016/S0140-6736(10)61889-2 PMC4176755

[88] Daley P, Jagannathan V, John KR, et al Adjunctive vitamin D for treatment of active tuberculosis in India: a randomised, double-blind, placebo-controlled trial. Lancet Infect Dis. 2015 5;15(5):528–534. 25863562 10.1016/S1473-3099(15)70053-8

[89] Tukvadze N, Sanikidze E, Kipiani M, et al High-dose vitamin D3 in adults with pulmonary tuberculosis: a double-blind randomized controlled trial. Am J Clin Nutr. 2015 11;102(5):1059–1069. 26399865 10.3945/ajcn.115.113886 PMC4625591

[90] Hasan Z, Salahuddin N, Rao N, et al Change in serum CXCL10 levels during anti-tuberculosis treatment depends on vitamin D status [Short Communication]. Int J Tuberc Lung Dis. 2014 4;18(4):466–469. 24670704 10.5588/ijtld.13.0460

[91] Salahuddin N, Ali F, Hasan Z, et al Vitamin D accelerates clinical recovery from tuberculosis: results of the SUCCINCT Study [Supplementary Cholecalciferol in recovery from tuberculosis]. A randomized, placebo-controlled, clinical trial of vitamin D supplementation in patients with pulmonary tuberculosis’. BMC Infect Dis. 2013 1 19;13:22. 23331510 10.1186/1471-2334-13-22 PMC3556334

[92] Ganmaa D, Giovannucci E, Bloom BR, et al Vitamin D, tuberculin skin test conversion, and latent tuberculosis in Mongolian school-age children: a randomized, double-blind, placebo-controlled feasibility trial. Am J Clin Nutr. 2012 8;96(2):391–396. 22760564 10.3945/ajcn.112.034967 PMC3396446

[93] Martineau AR, Wilkinson RJ, Wilkinson KA, et al A single dose of vitamin D enhances immunity to mycobacteria. Am J Respir Crit Care Med. 2007 7 15;176(2):208–213. 17463418 10.1164/rccm.200701-007OC

[94] Ralph AP, Waramori G, Pontororing GJ, et al L-arginine and vitamin D adjunctive therapies in pulmonary tuberculosis: a randomised, double-blind, placebo-controlled trial. PLoS One. 2013;8(8):e70032. 23967066 10.1371/journal.pone.0070032 PMC3743888

[95] Mily A, Rekha RS, Kamal SM, et al Significant effects of oral phenylbutyrate and vitamin D3 adjunctive therapy in pulmonary tuberculosis: A randomized controlled trial. PLoS One. 2015;10(9):e0138340. 26394045 10.1371/journal.pone.0138340 PMC4578887

[96] Xing Y, Liqi Z, Jian L, et al Doxycycline induces mitophagy and suppresses production of interferon-beta in IPEC-J2 cells. Front Cell Infect Microbiol. 2017;7:21. 28203548 10.3389/fcimb.2017.00021 PMC5285722

[97] Gao L, Tao Y, Zhang L, et al Vitamin D receptor genetic polymorphisms and tuberculosis: updated systematic review and meta-analysis. Int J Tuberc Lung Dis. 2010 1;14(1):15–23. 20003690

[98] Wallis RS, Zumla A Vitamin D as adjunctive host-directed therapy in tuberculosis: A systematic review. Open Forum Infect Dis. 2016 9;3(3):ofw151. 27800526 10.1093/ofid/ofw151 PMC5084719

[99] Dimitrov V, White JH Species-specific regulation of innate immunity by vitamin D signaling. J Steroid Biochem Mol Biol. 2016 11;164:246–253. 26369615 10.1016/j.jsbmb.2015.09.016

[100] Ponpuak M, Davis AS, Roberts EA, et al Delivery of cytosolic components by autophagic adaptor protein p62 endows autophagosomes with unique antimicrobial properties. Immunity. 2010 3 26;32(3):329–341. 20206555 10.1016/j.immuni.2010.02.009 PMC2846977

[101] Gomes MS, Paul S, Moreira AL, et al Survival of *Mycobacterium avium* and *Mycobacterium tuberculosis* in acidified vacuoles of murine macrophages. Infect Immun. 1999 7;67(7):3199–3206. 10377091 10.1128/iai.67.7.3199-3206.1999 PMC116496

[102] Luong K, Nguyen LT Impact of vitamin D in the treatment of tuberculosis. Am J Med Sci. 2011 6;341(6):493–498. 21289501 10.1097/MAJ.0b013e3182070f47

